# Immunotherapy for Hepatocellular Carcinoma: New Prospects for the Cancer Therapy

**DOI:** 10.3390/life11121355

**Published:** 2021-12-07

**Authors:** Rossella Fasano, Mahdi Abdoli Shadbad, Oronzo Brunetti, Antonella Argentiero, Angela Calabrese, Patrizia Nardulli, Roberto Calbi, Behzad Baradaran, Nicola Silvestris

**Affiliations:** 1 Medical Oncology Unit, IRCCS Istituto Tumori “Giovanni Paolo II”, 70124 Bari, Italy; r.fasano@oncologico.bari.it (R.F.); o.brunetti@oncologico.bari.it (O.B.); antonella.argentiero@oncologico.bari.it (A.A.); 2 Student Research Committee, Tabriz University of Medical Sciences, Tabriz 5165665811, Iran; abdolim@tbzmed.ac.ir; 3 Radiology Unit, IRCCS Istituto Tumori “Giovanni Paolo II”, 70124 Bari, Italy; a.calabrese@oncologico.bari.it; 4 Pharmacy Unit, IRCCS Istituto Tumori “Giovanni Paolo II”, 70124 Bari, Italy; p.nardulli@oncologico.bari.it; 5 Operative Unit of Radiology, Hospital Miulli, 70021 Acquaviva Delle Fonti, Italy; calbi.roberto@gmail.com; 6 Immunology Research Center, Tabriz University of Medical Sciences, Tabriz 5165665811, Iran; 7 Department of Immunology, Faculty of Medicine, Tabriz University of Medical Sciences, Tabriz 5166614766, Iran; 8 Department of Biomedical Sciences and Human Oncology, University of Bari “Aldo Moro”, 70124 Bari, Italy

**Keywords:** hepatocellular carcinoma, tumor microenvironment, immune cells, immunotherapy, single-cell sequencing

## Abstract

Hepatocellular carcinoma (HCC) is the fourth leading cause of cancer-related death worldwide. HCC patients may benefit from liver transplantation, hepatic resection, radiofrequency ablation, transcatheter arterial chemoembolization, and targeted therapies. The increased infiltration of immunosuppressive immune cells and the elevated expression of immunosuppressive factors in the HCC microenvironment are the main culprits of the immunosuppressive nature of the HCC milieu. The immunosuppressive tumor microenvironment can substantially attenuate antitumoral immune responses and facilitate the immune evasion of tumoral cells. Immunotherapy is an innovative treatment method that has been promising in treating HCC. Immune checkpoint inhibitors (ICIs), adoptive cell transfer (ACT), and cell-based (primarily dendritic cells) and non-cell-based vaccines are the most common immunotherapeutic approaches for HCC treatment. However, these therapeutic approaches have not generally induced robust antitumoral responses in clinical settings. To answer to this, growing evidence has characterized immune cell populations and delineated intercellular cross-talk using single-cell RNA sequencing (scRNA-seq) technologies. This review aims to discuss the various types of tumor-infiltrating immune cells and highlight their roles in HCC development. Besides, we discuss the recent advances in immunotherapeutic approaches for treating HCC, e.g., ICIs, dendritic cell (DC)-based vaccines, non-cell-based vaccines, oncolytic viruses (OVs), and ACT. Finally, we discuss the potentiality of scRNA-seq to improve the response rate of HCC patients to immunotherapeutic approaches.

## 1. Introduction

Hepatocellular carcinoma (HCC) is the most common malignant tumor of the liver, and it is the fourth leading cause of cancer-related death worldwide [1,2]. Chronic hepatitis mediated by the hepatitis B virus, alcohol abuse, hepatitis C infection, and steatohepatitis are the main risk factors of HCC development [3]. Metabolic diseases such as diabetes mellitus and obesity, along with smoking and genetic background, can increase the risk of HCC development [4]. Hepatocarcinogenesis is a sequential process characterized by chronic liver disease, fibrosis, and cirrhosis [5]. The early diagnosis of HCC allows for a wide range of therapeutic choices that improve affected patients’ overall survival (OS) and quality of life. Liver transplantation, hepatic resection, radiofrequency ablation (RFA), transcatheter arterial chemoembolization (TACE), and targeted therapies based on tyrosine protein kinase inhibitors are common therapeutic options for these patients. However, only one-third of the patients that are candidates for resection, transplantation, or local ablation experience median life lengths of more than 60 months [6]. HCC is an aggressive and progressive tumor. Indeed, 70–80% of advanced HCC patients do not benefit from these treatments due to late diagnosis [7,8]. Immunotherapy is an innovative therapeutic approach that has shown to be promising in treating various cancers. Preclinical and clinical studies have evaluated the safety and efficacy of immunotherapy in HCC. Immune checkpoint inhibitors (ICIs), adoptive cell transfer (ACT), and cell-based and non-cell-based vaccines are among the most commonly investigated therapeutic methods for HCC [9]. ICIs are monoclonal antibodies (mAbs) that selectively block the inhibitory immune checkpoints, e.g., programmed death-1 (PD-1), programmed death-ligand 1 (PD-L1), cytotoxic T-lymphocyte antigen 4 (CTLA-4), mucin domain molecule 3 (TIM-3), and lymphocyte activating gene 3 protein (LAG-3), so to enhance the T cell-mediated antitumoral immune responses [10]. Besides, cancer vaccines based on dendritic cells (DCs) or not based on cells, using a tumor-associated antigen (TAA), are promising approaches in HCC immunotherapy [9,11,12]. In the ACT, like chimeric antigen receptor T (CAR-T) cells, immune cells are engineered to express chimeric antigen receptors (CARs) so to identify and target specific cancer (neo-) antigens [13].

The tumor microenvironment (TME) has an essential role in determining the fate of the antitumoral immune responses. The immunosuppressive nature of the HCC microenvironment impedes the development of T cell-mediated antitumoral immune responses. The high level of immunosuppressive cytokines and the elevated expression of inhibitory immune checkpoints in the TME have been implicated in developing and maintaining the immunosuppressive milieu [14,15]. Besides, recent findings have indicated that subsets of immune cells, e.g., myeloid-derived suppressor cells (MDSCs), tumor-associated macrophages (TAMs), cancer-associated fibroblasts (CAFs), and regulatory T cells (Tregs), are other culprits in the development and maintenance of the immunosuppressive TME [16].

Recently, growing studies have characterized immune cell populations and identified intercellular cross-talk in the TME of HCC using single-cell RNA sequencing (scRNA-seq) technologies [17]. scRNA-seq are sophisticated technologies that have been effectively used in various research fields due to their ability to identify and analyze different cell types within tissues [18]. Since there is a vast inter- and intraheterogeneity in the tumor, scRNA-seq can effectively identify multiple subpopulations of tumoral cells [19]. Besides, scRNA-seq can categorize the phenotype of tumor-infiltrating immune cells and other cells residing in the TME, which can further our knowledge of the cross-talk in the tumor milieu [20].

Here, we aim to discuss the potentiality of immunotherapy in treating HCC. Besides, we shed light on the cross-talk between immune cells and tumor cells in the tumor microenvironment of HCC and highlight the recent findings obtained from the scRNA-seq of HCC.

## 2. HCC Microenvironment

In HCC development, the interactions between the tumoral cells and the tumor microenvironment are crucial. Hepatic fibrosis, hepatocarcinogenesis, epithelial–mesenchymal transition (EMT), invasion, and metastasis are all influenced by the TME. The main elements of the HCC tissue are cancer cells, innate immune cells, stromal cells, adaptive immune cells, endothelial cells, cancer-associated fibroblasts, and inflammatory cytokines. The interactions between the tumor-infiltrating effector cells and the other cellular and noncellular components of the TME are crucial in determining how HCC evolves [21,22].

Tumor cells and nonparenchymal cells, such as Kupffer cells (KCs), liver sinusoidal endothelial cells (LSECs), and hepatic stellate cells (HSCs) contribute to the creation of an immunosuppressive environment by expressing ligands that inhibit effector T and natural killer (NK) cell inhibitory receptors and secreting cytokines/chemokines that recruit tolerogenic cells (Tregs, MDSCs, macrophages, and neutrophils) [23].

The liver is thought to be an immunologically tolerant organ related to the unique physiological functions which it performs. Portal circulation exposes LSECs to a substantial number of antigens. These cells function as antigen-presenting cells (APCs) in the hepatic microenvironment, regulating immunogenicity. Their role in normal liver function is to inhibit an initial reaction to bacterial toxins so to avoid tissue damage [24]. As a result, the immunosuppressive molecules, including programmed cell death ligand-1, are expressed by LSECs (PD-L1) [25].

KCs, which are specialized liver-located macrophages that release immunosuppressive cytokines, such as IL-10 and prostaglandins, are another significant cell type [25]. They can also activate forkhead box P3 (FoxP3) in CD4^+^ T cells, causing CD4^+^ regulatory T cells (Tregs) to increase and negatively inhibit the immunological response. Therefore, compared to other cancers, HCC develops in a highly immunosuppressive environment which is responsible for tumor progression [26,27].

**Figure 1:**
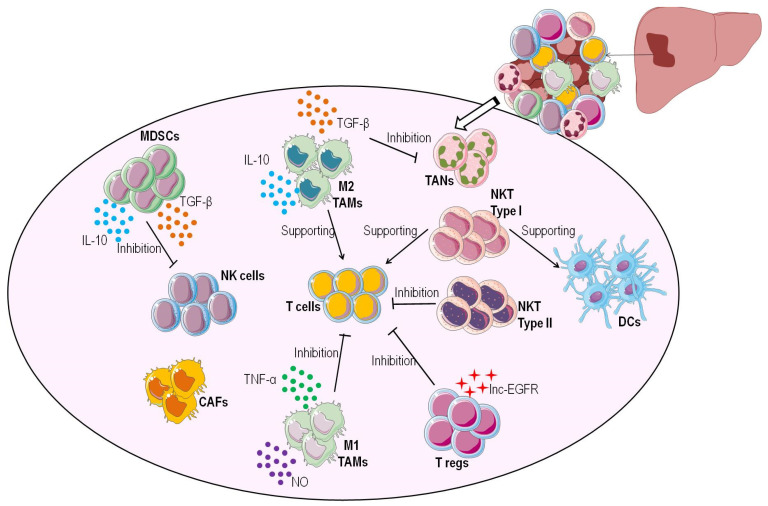
HCC immune microenvironment. The main elements of the HCC microenvironment are cancer cells, innate immune cells, stromal cells, adaptive immune cells, cancer-associated fibroblasts, and inflammatory cytokines. The interactions between tumor-infiltrating effector cells and other cell types are crucial in determining how HCC evolves. MDSCs release immunosuppressive cytokines such as IL-10 and TGF-beta, inhibiting NK cell antitumor activity. M1 TAMs releasing TNF-alpha and NO promote an antitumoral immune response. On the other hand, M2 TAMs producing IL-10 and TGF-beta inhibit immune response, leading to tumor development. The presence of TGF-beta in the tumor microenvironment suppresses degranulation by TANs, inhibiting their antitumor activity. Tregs overexpress lnc-EGFR, which reduces CTL activity, thus promoting HCC development. Type I NKT cells release proinflammatory cytokines, stimulating DCs and T cells; indeed, type II NKT cells suppress the immune response. DCs expressing inhibitory receptor ligands determine the downregulation of T cell-mediated immune response. CAFs contribute to developing an immunosuppressive microenvironment (http://smart.servier.com, accessed on 18 August 2021).

Moreover, tumor necrosis factor (TNF)-α and interleukin (IL)-12, inflammatory cytokines released from M1 macrophages, interferon (IFN)-γ expressed from T helper 1 (Th1), NK cells, and IL-2 secreted by cytotoxic T lymphocytes (CTLs) contribute to T cell activation [16]. However, one or more of these factors are compromised in HCC, leading to tumor development and metastasis [16]. In particular, the elevated expression of inhibitory immune checkpoints, e.g., CTLA-4 and PD-1, can substantially exhaust T cells and pave the way for the immune evasion of tumoral cells [14]. Furthermore, some populations of immune cells can enhance the immunosuppressive HCC microenvironment [16] (Figure 1).

## 3. Tumor-Infiltrating Immune Cells and Surrounding Cells

### 3.1. Myeloid-Derived Suppressor Cells

Myeloid-derived suppressor cells (MDSCs) are an immunosuppressive population present in local tissue and systemic circulation following inflammation or cancer [28,29]. They are primarily classified as CD11b^+^CD33^+^HLA^−^DR^-^ in humans; however, the coexpression of additional markers, e.g., CD14 and CD15, have also been observed to distinguish monocytic and granulocytic subsets [30]. Granulocyte colony-stimulating factor (G-CSF), granulocyte-macrophage colony-stimulating factor (GM-CSF), vascular endothelial growth factor (VEGF), monocyte chemoattractant protein-1 (MCP-1), and IL-1 are all tumor-derived cytokines that have been shown to facilitate MDSC infiltration into the TME [31].

Since MDSCs can release immunosuppressive factors, e.g., IL-10 and tumor growth factor (TGF)-β, augment inhibitory immune checkpoint signaling, and inhibit NK cell-mediated antitumoral immune responses, they pave the way for the immune evasion of the HCC cells [32]. Chiu et al. have shown that hypoxia in the tumor microenvironment of the HCC can pave the way for MDSC infiltration via the hypoxia-inducible factor (HIF)-dependent regulation of the CCL26/C-X3-C motif chemokine receptor 1 (CX3CR1) pathway [33]. Furthermore, IL-6, IL-1β, VEGF, GM-CSF, and G-CSF can contribute to MDSC infiltration in the tumor microenvironment [31]. Besides, MDSCs can express galectin 9 (Gal-9) and facilitate T cell apoptosis and the immune evasion of HCC cells [34]. It has been demonstrated that the coculture of MDSCs with NK cells can remarkably decrease the cytotoxicity of NK cells. The MDSC-mediated suppression of the NK cells is mainly dependent on NKp30 on the NK cells. Therefore, inhibiting MDSCs can substantially simulate antitumoral immune responses [32].

### 3.2. Tumor-Associated Macrophages

Tumor-associated macrophages (TAMs) are among the primary tumor-infiltrating immune cells in the tumor microenvironment and substantially contribute to the HCC development [35]. Various chemokines, e.g., chemokine (CCL) 2, CCL5, CCL7, CX3CL, and cytokines such as macrophage colony-stimulating factor (M-CSF), GM-CSF, and VEGF can facilitate monocyte infiltration into the tumor microenvironment, leading to TAMs development [36]. M1 and M2 are the two groups of TAMs that play different roles in tumor development. Interferon (INF)-α, INF-β, and INF-γ can activate M1 macrophages; however, IL-4 and IL-10 activate M2 macrophages. M1 cells can stimulate antitumoral immune responses by releasing TNF-α and nitric oxide; however, M2 cells can express VEGF, IL-10, TGF-β, and indoleamine 2,3-dioxygenase (IDO), leading to tumor development [16,37,38].

Dong et al. have shown that CD68^+^ TAMs alone are not correlated with the clinicopathological characteristics and prognoses of HCC patients. Nevertheless, M1 cells express high levels of CD86, TNF-α, and IL-12, while M2 cells overexpress CD206, CD163, IL-10, and TGF-β. The low infiltration of CD86^+^ cells (M1) and the increased infiltration of CD206^+^ (M2) TAMs have been associated with aggressive tumor phenotypes. Therefore, the CD86^+^/CD206^+^ ratio can confer valuable insights into the phenotype of the HCC cells [35].

### 3.3. Tumor-Associated Neutrophils

Tumor-associated neutrophils (TANs) have different functions in several cancer types, e.g., lung cancer, gastric cancer, and lymphoma [39,40,41]. TANs can display antitumorigenic (N1) or protumorigenic (N2) phenotypes, and the presence of TGF-β relies on the plasticity of these subtypes [42,43]. In particular, the presence of TGF-β can lead to N2 cell development, and the inhibition of TGF-β or the presence of type-I IFNs can result in N1 cell development [44,45]. When the neutrophils are exposed to lipopolysaccharide (LPS), TGF-β has been shown to suppress degranulation while inhibiting the release of reactive oxygen species (ROS), reactive nitrogen intermediates, and IL-1β by TANs. TGF-β signaling inhibition, on the other hand, was found to confer antitumorigenic activity to neutrophils, which was associated with alterations in chemokine and cytokine profiles, the increased neutrophil infiltration of tumors, and increased intercellular adhesion molecule 1 (ICAM1) expression on the endothelial cells. Type I interferon-deficient neutrophils formed fewer neutrophil extracellular traps (NETs) and exhibited a worse ability to kill tumor cells than their interferon-producing counterparts. The presence of these conditions was associated with a significant increase in neutrophil turnover and an increase in immature neutrophils [45,46].

Multiple studies have shown that the phenotype of the neutrophils in HCC is predominantly N2. He et al. have demonstrated that the high expression of GM-CSF and TNF-α in the peritumoral area of HCC can increase CD66b, PD-L1, TNF-α, and CCL2 expression via modulating the neutrophils to adopt an immunosuppressive profile. Indeed, the immunosuppressive neutrophils can induce CD8^+^ T cell apoptosis [47,48].

Zhou et al. have shown that the microRNA (miR)-301b-3p overexpression on the HCC cells can stimulate nuclear factor kappa-light-chain-enhancer of activated B cell (NF-kB) signaling and CXCL5 expression. CXCL5 expression on the HCC cells had a chemoattractant effect on the neutrophils determining the TANs infiltration. However, the direct interaction between the TANs and the HCC cells has not been clarified yet, but it has been shown that it correlates with poor prognoses in patients with HCC. CXCL5 acts as a prognostic factor for HCC patients’ overall survival [49,50]. Besides, Li et al. have reported that the presence of intratumoral CD66b^+^ neutrophils is strongly associated with advanced Barcelona Clinic Liver Cancer (BCLC) stage, liver fibrosis, elevated serum gamma-glutamyl transferase (γ-GT), and worsened progression-free survival (PFS) and OS [51].

In contrast, IFN-I paves the way for developing antitumoral TANs (N1). It has been shown that inhibiting the TGF-β signaling can lead to the recruitment and activation of CD8^+^ T cells, macrophages, and N1 neutrophils [46,52].

### 3.4. Regulatory T Cells

Regulatory T cells (Tregs) are a subset of CD4^+^ T cells that express CD25 and forkhead box P3 (FoxP3). Tregs are primarily responsible for suppressing immune response; therefore, their decreased level is associated with an increased risk of autoimmunity and allergic reactions [53]. Tregs can substantially suppress the antitumoral immune responses in the tumor microenvironment, paving the way for the immune evasion of the tumoral cells [16,54]. T cell receptor (TCR) interaction with IL-10 and TGF-β signaling facilitates the infiltration of Tregs into the tumor microenvironment by regulating the CCL6/CCL20 axis [16]. Jiang et al. have shown that the Tregs-overexpressed long noncoding RNA (lnc)-epidermal growth factor receptor (EGFR) can induce Treg differentiation, attenuate CTL activity, and promote HCC development. LncRNAs are a novel class of non-protein-coding transcripts that play a critical role in the development of human carcinoma [55,56]. In particular, in HCC, to understand how it works, lnc-EGFR specifically interacts with EGFR and prevents it from interacting with and being ubiquitinated by the cell death protein (c-CBL), thereby stabilizing it and increasing the activation of itself and the downstream activator protein-1 (AP-1)/nuclear factor of activated T cell-1 (NF-AT1) axis, which in turn induces EGFR expression [57].

### 3.5. CD8^+^ Cytotoxic T Lymphocytes

CD8^+^ cytotoxic T lymphocytes (CTLs) are a subset of T cells expressing CD8 on the surface and which target tumoral cells. Specifically, CTL-secreting perforin, granzyme, and TNF-α can eradicate cancer cells [16].

Despite the fact that the antitumor immune response is severely repressed by various mechanisms in HCC, the presence of CD8^+^ CTLs in the tumor is associated with increased survival. Tumor-associated antigen (TAA)-specific response and IFN-γ production by CTLs are restricted due to hypoxia, metabolic competition with cancer cells (HCCs), a deficiency in CD4^+^ T cells (CD4+ T cells are lacking), and the high expression of several regulatory molecules (VEGF, CXCL17, IDO, and IL-10) in the tumor microenvironment [16].

It has also been shown that the number of CTLs in HCC patients is increased; however, they cannot kill the tumoral cells. Guo et al. have demonstrated that CD8^+^ T cells express high levels of Fas that interact with the Fas ligand (FasL), leading to CD8^+^ T cell apoptosis in HCC patients [58].

CD14^+^ DCs, a subpopulation of DCs present in the blood of HCC patients, can release large amounts of IL-10 in response to LPS, and neutralizing the anti-IL-10 mAb can significantly reduce the immune-suppressive activity of CD14^+^DCs, thus implying that they suppress T cell responses in part through IL-10 production. Han Y. et al. have reaffirmed the critical function of IL-10 in tumor immunosuppression [59]. Additionally, IDO has been implicated in Tregs as an immunosuppressive effector mechanism [60]. Tregs can induce DCs to produce IDO, which therefore suppresses T cell responses indirectly [61]. They also discovered that CD14^+^ DCs express large levels of IDO in the absence of LPS stimulation, and that the IDO inhibitor metallothionein 1 (MT-1) almost completely reduced the immunoregulatory function, indicating that IDO may be more essential than IL-10 in mediating the suppressive action of CD14^+^ DCs [59].

Although the CTLs can develop robust antitumoral immune responses, the immunosuppressive tumor microenvironment can substantially exhaust the CTLs [62,63].

### 3.6. Natural Killer Cells

Natural killer (NK) cells can mediate antitumoral immune responses via various mechanisms. In the “missing self-response” mechanism, NK cells detect the downregulation of major histocompatibility complex (MHC) class I [64,65]. In the “altered self-response” mechanism, NK cells detect the high expression of cell stress ligands [66,67]. Besides, NK cells can derive antitumoral immune responses via the antibody-dependent cellular cytotoxicity (ADCC) mechanism [68]. Although there is a high level of tumor-infiltrating NK cells in HCC, their antitumoral immune responses are limited [69,70].

NK cells can be categorized into two subsets, i.e., CD56^bright^ and CD56^dim^. The CD56^bright^ NK cell subset is characterized by producing many cytokines and limited cytotoxic properties. The level of CD56^bright^ NK cells in the human liver is much higher than in the peripheral blood [71,72,73]. On the other hand, CD56^dim^ NK cells are the majority of the circulating NK cells that generate cytokines and that have cytotoxic properties. Cai et al. have reported a remarkable reduction in the level of CD56^dim^ NK cells in HCC tissues compared to nontumoral tissues. Because NK cells can target the tumoral cells via the ADCC mechanism, the decreased level of the NK cells can reduce the cytotoxic activity of the NK cells [73]. 

The advantage of the NK cells is that they can release cytolytic granules directly to target the tumor cells and express proinflammatory factors to stimulate the antitumoral immune response. However, the immunosuppressive tumor microenvironment can substantially inhibit the antitumoral immune responses of NK cells [74,75].

### 3.7. Invariant Natural Killer T Cells

Invariant natural killer T (iNKT) cells play a pivotal role in regulating the innate and adaptive immune systems. The iNKT cells release cytokines that can alter the polarization and activation of immune cells, especially NK and T cells. Indeed, they are a unique sub-population of mature CD4^+^ T cells that can simultaneously express both NK cell surface markers and TCRs [76,77].

As a member of the main MHC class I-like family, CD1d is abundant in hepatocytes. It can stimulate NKT cell development and activity. CD1d-restricted NKT cells are divided into two subgroups, i.e., type I and type II. Type I NKT cells recognize self-lipids and microbial lipid antigens. Activated type I NKT cells release proinflammatory cytokines that stimulate pit cells, lymphocytes, and DCs [77]. On the other hand, type II NKT cells can release cytokines that suppress immune responses [77,78]. 

NKT cells have a role in the pathophysiology of cirrhosis via producing cytokines implicated in fibrosis development. Although there have been a few investigations, intratumoral NKT cells can retain the ability to destroy HCC cells [79,80]. It has been shown that the level of NKT is substantially decreased in HCC [81].

### 3.8. Dendritic Cells

Dendritic cells (DCs) are antigen-presenting cells that can activate T cells. The liver contains various DCs, including progenitor, immature, and mature cells [82]. When DCs pick up antigens, they migrate to a regional lymph node where they become mature and deliver the antigen to naïve T cells. Resident hepatic DCs are found around the central and portal veins and are originated from the bone marrow [76].

In carcinogenesis and viral infection, DCs play a critical role. DCs contribute to tumor development by two primary mechanisms: tumor antigen tolerance and inhibiting T cell activity through the release of mediators or the expression of checkpoint ligands. Although most of these mechanisms have been identified in human HCC samples, there is only limited evidence on the lipid overload-mediated DC dysfunction [83]. Although mature the DCs can activate the T cell-mediated antitumoral immune responses, they can also pave the way for HCC development via inducing tolerance. Through increased IL-10 and low IL-12 signaling, mature DCs can produce Treg1-like cells from naïve CD4^+^ T cells [83].

In HCC, the DCs express inhibitory receptor ligands for PD-1, TIM-3, LAG-3, and CTLA-4. Besides, these inhibitory immune checkpoints are substantially upregulated in the CD4^+^ and CD8^+^ T cells of HCC patients, determining the downregulation of T cell-mediated immune response [84].

### 3.9. Cancer-Associated Fibroblasts

Fibroblasts are the cells involved in the formation of the extracellular matrix. In particular, cancer-associated fibroblasts (CAFs) play a critical role in cancer development by remolding the extracellular matrix and releasing soluble factors and exosomes [16,85,86]. Besides, CAFs attract immune cells, e.g., DCs, neutrophils, and monocytes, and contribute to developing an immunosuppressive microenvironment in the HCC [87,88]. CAFs can be originated from activated mesenchymal stem cells. Zhou et al. have demonstrated that tumor-derived miR-21-5p might convert HSCs to CAFs by downregulating phosphatase and the tensin homolog (PTEN), resulting in the phosphoinositide-dependent kinase-1 (PDK1)/AKT signaling pathway stimulation. Of interest, CAFs can secrete protumoral factors, e.g., VEGF, fibroblast growth factor (FGF), TGF-β, matrix metalloproteinase (MMP)2, and MMP9. Moreover, a high level of miR-2-5p in serum has been associated with the poor prognosis of HCC patients [16,89].

### 3.10. Liver Sinusoid Endhotelial Cells

Liver sinusoid endothelial cells (LSECs) are the most common nonparenchymal cell type in the liver, accounting for 15–20 percent of total liver cells [90,91]. LSECs are particular endothelial cells with a minimal basement membrane and with fenestrations. These characteristics make them the most permeable endothelial cells in mammals [90,92]. LSECs play an important role in both innate and adaptive immunity, as well as in immunological tolerance maintenance in the liver [93,94]. Because of their particular characteristics and functions, LSECs can contribute to making the tumor microenvironment immunosuppressive and support the development of liver cancer [95]. LSECs can cross-present soluble antigens to CD8^+^ T cells via their MHC-I. CD8^+^ T cells that have been stimulated by LSECs recover a quiescent condition, unable to exert cytotoxic effects on tumor cells due to coinhibitory signaling [96]. Furthermore, it has been proven that LSECs can induce tumor cell tolerance [97]. Another way that the liver sinusoids could be protumorigenic is through the TGFβ-dependent Treg activation [98].

### 3.11. Kupffer Cells

Kupffer cells (KCs) are tissue macrophages found in the liver sinusoids’ lumen and represent the first line of defense against pathogens [99]. Toll-like receptor expression activates KCs in response to endotoxins, complements, and other pathogen-associated molecular patterns. The KCs release a variety of cytokines and chemokines, which stimulate neutrophils [100]. The KCs are also the first line of defense against cancer cells that have spread to other organs and infections. The KCs, releasing PD-L1, inhibit the cytotoxic function of CD8^+^ T cells [101]. In addition, KC release of IL-6 promotes the development and progression of HCC [102]. When stimulated by inflammatory cytokines (IL-1, TNF alpha, and PDGF), the KCs and HSCs secrete abundant osteopontins, which play a key role in many cell-signaling pathways that promote inflammation, tumor growth, and metastasis [103].

### 3.12. Hepatic Stellate Cells

Hepatic stellate cells (HSCs) are found in the Disse space, between LSECs and hepatocytes, and are involved in the development of liver fibrosis, which can eventually lead to HCC [104]. Activated HSCs have been linked to an immunosuppressive environment in patients with HCC, as well as a poor clinical prognosis [105,106]. When activated, the HSCs shift monocytes from an inflammatory to an immunosuppressive state and support HCC proliferation [105]. Furthermore, they promote the accumulation of MDSC in a contact-dependent manner, either through the CD44 expression on HSC or hydrogen peroxide depletion by catalase [97,107]. HSCs can also cause T cell dysfunction directly by the expression of PD-L1 and the limiting of T cell growth and function by expressing TGF-β [108,109].

## 4. The Role of Inhibitory Immune Checkpoints in HCC Development

**Figure 2:**
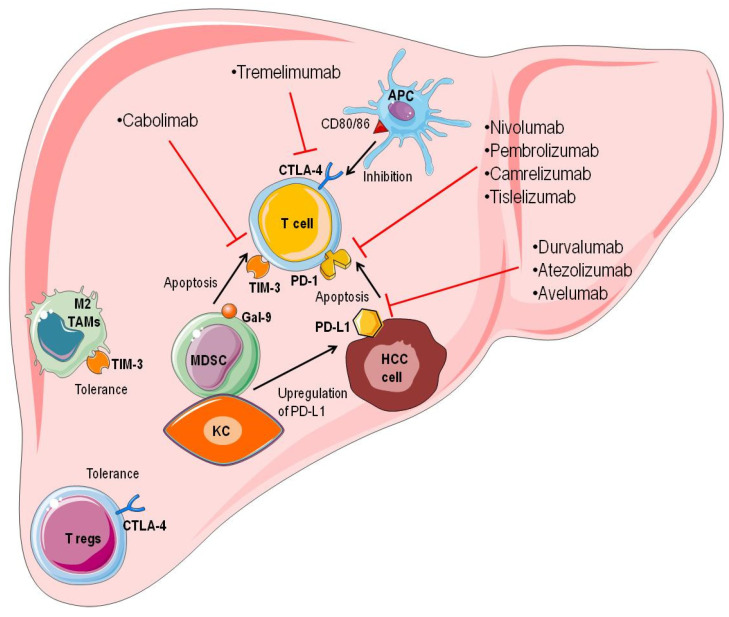
Immune checkpoints and their inhibitors. HCC has an immunosuppressive tumor microenvironment. Inhibitory immunological checkpoints are one of the most critical components in the development of an immunosuppressive milieu. Indeed, elevated levels of PD-1, CTLA-4, LAG-3, and TIM-3 expression in the tumor microenvironment have been linked to reduced T cell-mediated antitumor immune responses. The interaction of PD-1 on tumor-infiltrating CD8^+^T cells and its ligand, PD-L1, on tumoral cells can lead to CD8^+^ T cell apoptosis. Moreover, MDSCs may interact with KCs to upregulate PD-L1 expression in HCC cells. CTLA-4 is expressed on activated T cells and Tregs, and it has a high affinity for competing with CD28 on APCs by binding to its ligands, CD80 and CD86. CTLA-4 inhibits the proliferation of T cells. MDSCs can express Gal-9, which interacts with Tim-3 on T cells, determining T cell apoptosis. TIM-3 is also expressed on TAMs, facilitating M2 polarization and HCC development. Inhibitory immune checkpoint molecules (ICIs) can pave the way for the immune evasion of tumoral cells. Nivolumab, pembrolizumab, camrelizumab, and tislelizumab are anti-PD-1 antibodies. Durvalumab, atezolizumab, and avelumab are anti-PD-L1 antibodies. Tremelimumab is an anti-CTLA-4 antibody, and cobolimab is an anti-Tim-3 antibody. These molecules can liberate T cells from exhaustion and facilitate the development of antitumoral immune responses (http://smart.servier.com, accessed on 20 August 2021).

As discussed above, the tumor microenvironment of HCC is immunosuppressive. Inhibitory immune checkpoints are among the critical factors that contribute to the development of the immunosuppressive milieu. Indeed, the high expression of PD-1, CTLA-4, LAG-3, and TIM-3 in the tumor microenvironment has been associated with the attenuated T cell-mediated antitumoral immune responses [84,110] (Figure 2).

### 4.1. Programmed Death-1/Programmed Death Ligand-1

The programmed death-1 (PD-1)/programmed death ligand-1 (PD-L1) axis plays a critical role in maintaining peripheral tolerance [111,112]. Growing evidence indicates that the PD-L1/PD-1 axis can exert an inhibitory signal to PD-1-expressing T cells, causing the exhaustion of T cells [113]. Consistent with this, recent findings have demonstrated that the activation of the PD-1/PD-L1 axis can substantially attenuate the antitumoral immune responses. Therefore, blocking this axis with the pertained mAbs can be considered a therapeutic approach for HCC patients [114].

The interaction of PD-1 on tumor-infiltrating CD8^+^ T cells and its ligand, PD-L1, on tumoral cells can lead to CD8^+^ T cell apoptosis. The PD-1 upregulation on the circulating and tumor-infiltrating CD8^+^ T cells might be associated with the inferior survival of affected patients and tumor relapse after resection in HCC patients. In particular, Shi et al. have shown that PD-1 expression is substantially elevated in the peripheral and tumor-infiltrating CD8^+^ T cells in HCC patients [114]. Moreover, MDSCs may interact with KCs to upregulate PD-L1 expression in patients with advanced HCC [32]. Furthermore, type I or type II interferon can remarkably increase PD-L1 expression, resulting in tumor development, vascular invasion, and poor prognosis in HCC patients [115,116]. In the last years, it has been shown that differential PD1 expression levels on CD8^+^ T cells exist in HCC patients, and this allows for a phenotypic and functional classification of the tumor-infiltrating CD8^+^ T cells. Based on PD-1 expression, the exhausted CD8^+^ T cells can be distinguished into three subpopulations: PD1-high, PD1-intermediate, and PD1-negative cells. The gene expression profiles of these subpopulations are different. In particular, T cell exhaustion genes are expressed at higher levels in PD1-high cells, and in TIM-3 and LAG-3, than in PD1-intermediate cells [117].

### 4.2. Cytotoxic T-Lymphocyte Antigen 4

Cytotoxic T-lymphocyte antigen 4 (CTLA-4) is another inhibitory immune checkpoint molecule that is expressed on activated T cells and Tregs [118,119]. CTLA-4 has a high affinity for competing with CD28 on APCs by bindings to its ligands, i.e., CD80 and CD86. CTLA-4 can substantially regulate CD4^+^ T cell function and inhibit the proliferation of T cells. Besides, CTLA-4 contributes to the development of the immunosuppressive tumor microenvironment in HCC via promoting Treg, IDO, and IL-10 production in the DCs [59,120]. 

### 4.3. Mucin Domain Molecule 3

Mucin domain molecule 3 (TIM-3) is well known for its inhibitory function on tumor-infiltrating lymphocytes (TILs). The Tim-3 ligands are galectin-9 (Gal-9), phosphatidylserine (PtdSer), high mobility group box-1 protein (HMGB1), and carcinoembryonic antigen-related cell adhesion molecule 1 (CEACAM-1) [121]. When TIM-3 interacts with Gal-9, the activation of the TIM-3/Gal-9 axis can predict the inferior survival of the HBV-related HCC patients [122]. MDSCs can also express Gal-9, which can result in T cell apoptosis [34]. Besides, tumor-intrinsic TIM-3 can activate the EMT process and increase tumor growth. On the other hand, TIM-3 ligands are widely expressed throughout the tumor microenvironment, potentially mediating the interaction between the tumor cells and the nonparenchymal cells, thereby influencing the aggressive phenotype of the HCC cells [121]. It has been shown that TIM-3 is also expressed on TAMs, facilitating M2 polarization and HCC development [123]. Furthermore, TIM-3-expressing macrophages cannot phagocytize apoptotic bodies, causing the equilibrium of the tumor microenvironment to be further disrupted in HCC [124,125]. These findings indicate that targeting TIM-3 can stimulate antitumoral immune responses [121].

### 4.4. Lymphocyte Activating Gene 3

Lymphocyte activating gene 3 (LAG-3) is an immunoglobulin super family protein that can bind to MHC class II molecules with a high affinity [126]. Recently, it has been shown that LAG-3 plays an immunosuppressive role in chronic viral hepatitis and HCC [9].

## 5. Immunotherapeutic Approaches for Treating HCC

Many clinical trials, including ICIs, cancer vaccines, ACT, and combinations with chemo-radiotherapy or other molecularly targeted treatments evaluate the immunotherapeutic efficacy in HCC with some promising results [13].

The combination of bevacizumab (an anti-VEGF) plus atezolizumab (an anti-PD-L1) is the only immunotherapeutic approach used as a first-line treatment in HCC patients with portal invasion, extrahepatic spread, preserved liver function, and ECOG PS 0–2 [127]. With the improvement of our knowledge in cancer immunotherapy, in the future immunotherapy might be a standard therapeutic option for HCC. In other tumors, for example, in lung cancer, the United States Food and Drug Administration and the European Medicines Association have approved pembrolizumab (an anti-PD-1) as a first-line treatment in patients with metastatic lung cancer whose tumors have a PD-L1 expression of ≥50% with no other genomic tumor aberrations [128] (Table 1).

### 5.1. Immune-Checkpoint Inhibitors

Inhibitory immune checkpoint molecules (ICIs) can pave the way for the immune evasion of tumoral cells. ICIs have shown promising results in treating HCC [110]. Nivolumab is an anti-PD-1 that liberates T cells from exhaustion and facilitates the development of antitumoral immune responses. Based on the results of the CheckMate 040, the Food and Drug Administration (FDA) granted an accelerated approval to nivolumab for advanced HCC patients who have been previously treated with sorafenib (NCT01658878). This was a multicenter, open-label, phase I/II, noncomparative, dose escalation, and expansion trial. Patients were given a drug dose of 0.1–10 mg/kg every two weeks during the dose escalation phase. Patients were given a medication dose of 3 mg/kg once every two weeks during the dose expansion phase. Nivolumab had a manageable safety profile in both phases, with a good tolerability and an incidence of treatment-related adverse events that were not dose-related. Furthermore, the dose expansion phase demonstrated the long-term objective responses, with 15–20 percent objective response rates, a median overall survival of 15.6 months, and a considerable tumor decrease [129].

Pembrolizumab is another anti-PD-1 that has been tested in the KEYNOTE-224 trial (NCT02702414). This was a phase II, single-armed, nonrandomized study. Patients were given 200 mg of pembrolizumab intravenously every three weeks for two years or until progression. The objective response rate was 17%, with one patient (1%) achieving a complete response, 17 patients (16%) achieving partial responses, 46 patients (44%) achieving stable disease, and 34 patients (33%) with progression [130].

Camrelizumab is an anti-PD-1 that binds to a different epitope compared to nivolumab and pembrolizumab. The efficacy and safety of camrelizumab were investigated for HCC patients in the phase II trial (NCT02989922). This was a multicenter, open-label, parallel-grouped, randomized phase II trial. The findings showed that camrelizumab has manageable drug toxicity, a 14.7 percent objective response, and a 74.4 percent and 55.9 percent overall survival probability at 6 and 12 months, respectively, suggesting that it could be used as a new second-line therapeutic option for patients with advanced HCC [131]. Tislelizumab is another anti-PD-1 that is being investigated as a first-line treatment in patients with advanced unresectable HCC in the RATIONALE-301 trial (NCT03412773). This was a multicenter, randomized, open-label trial comparing the efficacy and safety of tislelizumab versus sorafenib as a first-line treatment in patients with advanced unresectable HCC [131].

A clinical trial (NCT01693562) has shown that durvalumab, as an anti-PD-L1, can demonstrate an acceptable safety profile and exert promising antitumoral responses [132]. Atezolizumab and avelumab are other anti-PD-L1s that are being investigated for treating HCC patients [142]. Tremelimumab is the first completely human immunoglobulin (Ig)G2 mAb that is being studied to target CTLA-4 in HCC patients (NCT01008358). In this pilot, the clinical trial was to evaluate the antitumor and antiviral effects of tremelimumab. Tremelimumab (15 mg/kg IV) was given every 90 days until the tumor advanced or until significant toxicity developed. The toxicity and viral response were assessed in 20 patients, while the tumor response was evaluated in 17 cases. The findings showed a partial response rate of 17.6% and a disease control rate of 76.4%. The time to progression was 6.48 months. The study has shown the good safety profile of tremelimumab [133]. 

Cabolimab (TSR-022) is an anti-TIM-3 that is being tested in patients with advanced solid tumors, including HCC (NCT02817633) [110].

### 5.2. Dendritic Cell-Based Vaccines

Dendritic cells (DCs) have recently attracted growing attention for treating solid tumors such as HCC. Accumulating in vitro and in vivo studies have identified some tumor-associated antigens (TAAs) for generating DC vaccines [9,11].

In 2003, the first clinical experiment was designed to stimulate specific T cell responses against the alpha-fetoprotein (AFP). AFP is a self-protein that is highly produced in the fetal liver; however, its level in the serum of adults is, physiologically, around 5 to 10 μg/L. HCC cells upregulate the AFP, and patients with active disease may have plasma levels as high as 0.5–1 mg/mL [143,144]. Butterfield et al. have performed a phase I/II trial in which AFP-positive HCC patients were immunized with DC vaccines against AFP peptides. Ten patients with HCC were treated. Six of the ten patients had statistically significant increases in the AFP-specific T cells after vaccination to at least one peptide by MHC tetramer. In addition, six of the ten subjects increased IFN-γ after vaccination, resulting in AFP-specific T cell responses to at least one of the peptides. They have shown that, following the infusion of the AFP peptide-pulsed DCs, the T cells can respond to the AFP in the presence of large quantities of this oncofetal antigen in the blood. Although this vaccine was an effective immunologic stimulus, objective clinical responses were not observed in this group of HCC patients [145]. Then, vaccines based on autologous DCs pulsed ex vivo with either an autologous tumor lysate or the lysate of HepG2 cells were developed [134,142,146].

Cancer-testis antigens (CTAs) are also promising targets for HCC immunotherapy [147]. New York esophageal squamous cell carcinoma-1 (NY-ESO-1), also known as CTAG1, is a CTA that is highly immunogenic. Earlier studies have found that NY-ESO-1 is strongly expressed in various solid tumors, and DC vaccines targeting NY-ESO-1 were investigated [148]. NY-ESO-1 expression can facilitate tumor development and the DCs loaded with the NY-ESO-1 peptide can trigger specific T cell responses against the HCC cells [137].

### 5.3. Non-Cell-Based Vaccines

Following the discovery of TAAs, vaccines targeting the TAAs have been developed. Tumor antigens, such as glypican-3 (GPC-3), and telomerase reverse transcriptase (hTERT) have been identified as potential vaccine-based immunotherapeutic targets for HCC [12].

Clinical evidence on the GPC-3-based vaccines suggests that they can produce antitumoral immune responses, and that their administration is associated with the improved OS of HCC patients [135,149]. Consistent with this, it has been reported that GPC3 is overexpressed in HCC cells and is associated with the worsened prognosis of HCC patients [150]. Sawada et al., in a nonrandomized, open-label, phase I clinical trial have analyzed the safety and efficacy of GPC3 peptide vaccination in patients with advanced HCC. In this study, thirty-three patients with advanced HCC were enrolled. The GPC3 vaccine was well tolerated. One patient had a partial response, and nineteen patients had a stable disease two months after the treatment began. Four of the nineteen patients with the stable disease had tumor necrosis or regression. In nine patients, the levels of the tumor markers, such as α-fetoprotein, temporarily decreased. In 30 patients, the GPC3 peptide vaccine elicited a GPC3-specific CTL response. Furthermore, the patients with high GPC3-specific CTL frequencies had a significantly longer OS than those with low frequencies. It has been shown that the vaccination against the two GPC3 peptides can result in specific CTL responses in patients with advanced HCCs [135].

As a catalytic enzyme essential for telomere elongation, hTERT is overexpressed in 80–90% of HCC cells. Mizukoshi et al. have identified human leukocyte antigen (HLA)-A*2402-restricted T cell epitopes derived from hTERT, and subsequently examined the hTERT-specific immunological responses in HCC patients. Positive T cell responses were seen in 6.9 to 12.5 percent of HCC patients. Even in patients with the early stages of HCC, hTERT-specific T cell responses were observed. In conclusion, the peptides containing the epitopes have generated hTERT-specific CD8^+^ T cell-mediated immune responses and exhibited a high affinity for binding to HLA-A*2402. Indeed, this enzyme represents a promising target in HCC immunotherapy [138].

A multiepitope and multi-HLA peptide vaccine is currently being investigated for safety and immunogenicity in HCC patients undergoing surgical and/or locoregional treatments [25]. A single-arm, open-label, multicenter, first-in-man phase I/II study (NCT03203005) evaluated a multi-peptide-based HCC vaccine (IMA970A) plus a CV8102 adjuvant (RNAdjuvant) in patients with very early, early, and intermediate-stage HCC. This trial is currently ongoing to assess the safety and immunogenicity of this vaccination [151].

### 5.4. Oncolytic Viruses

Since oncolytic viruses (OVs) can preferentially replicate in the tumoral cells and can infect the tumoral cells throughout the tumor bulk, they are promising strategies for cancer immunotherapy [152]. Despite their advantages, OV dosing must be adequately specified for clinical use; therefore, more clinical trials are needed to answer these questions [9].

Recently, a herpes simplex virus type 1-based oncolytic vector, LDO-GFP, has been introduced as a new agent to destroy HCC cells. The oncolytic activity of LDO-GFP against HCC was examined in vitro and in vivo, as well as the safety profile of LDO-GFP in immunocompetent mice. Such a safe and effective OV appears to be a suitable option for people with HCC. The intratumoral and intravenous administration of LDO-GFP has been associated with decreased tumor growth without substantial toxicity [139].

Another group has investigated the antitumoral effect of Golgi protein 73 (GP73)-sphingosine kinase 1 (SphK1)-short RNA (Sr)-adenovirus serotype 5 (Ad5) on the development of HCC. GP73-SphK1sR-Ad5 was produced by integrating the GP73 promoter and SphK1-shRNA into Ad5 and transfected into HCC Huh7 cells and normal human liver HL-7702 cells. This construct has displayed an inhibitory effect in HCC Huh7 cells and stimulated the expression of adenovirus early region 1A (E1A), a gene involved in viral replication in host cells but not in HL-7702 cells. These results have shown that GP73-SphK1sR-Ad5 is exclusive for the HCC cells. In animal models, its intratumoral injection has decreased the tumor volume, the tumor infiltration area, blood vessel density, and prolonged the survival of the affected animals [140].

Cytokine-armed vaccinia virus can also effectively inhibit the development of HCC. Zhang et al. have developed a cancer-targeted vaccinia virus containing the IL-37 gene knocked in the region of the viral thymidine kinase (TK) gene, called VV-IL-37. In vitro results have shown that VV-IL-37-infected HCC cells demonstrate decreased proliferation, migration, and invasion via the reduction of the signal transducer and the activator of transcription 3 (STAT3) phosphorylation. In vivo results have demonstrated that VV-IL-37 can increase IL-37 expression and stimulate antitumoral immune responses [141].

### 5.5. Adoptive Cell Transfer

Adoptive cell transfer (ACT) is a highly personalized cancer treatment. Indeed, the recent advances in cell therapy have allowed us to modify lymphocytes to express chimeric antigen receptors (CARs). CARs are synthetic receptors that allow T lymphocytes to identify TAAs in an MHC-independent manner [153]. Ongoing clinical trials are investigating the therapeutic potentiality of CAR-T cells in treating advanced HCC. An open-label, single-arm pilot study (NCT03130712) evaluated anti-GPC3 CAR T cells in patients with advanced HCC. For this purpose, patients were subjected to leukapheresis to obtain PBMCs. Activated T cells were engineered to express CARs that are specific to GPC3. These cells were expanded and administrated to the patient. In four advanced HCC patients, two escalating dosage levels (DLs) of CAR-T (1 × 10^5^ to 5 × 10^5^ CAR+ T cells/cm^3^) were used. Injections were made from three angles at two regions of the tumor under ultrasound supervision. In vivo, the GPC3-CAR-T cells expanded and were found in the peripheral blood of individuals who had a positive response. On days 12–15 after the intratumor injection of the CAR-T cells, three of the four patients had grade 2 cytokine release syndrome, but the tumor marker AFP in the blood did not change significantly. Two of the four patients who had previously suffered progressing disease (PD) developed the stable disease (SD) for more than 12 weeks. One patient had a partial response (PR) after 4 weeks, with portal vein tumor thrombus, but his body started producing the anti-GPC3 antibody after the second and third injections at 6 and 14 weeks, respectively, and he developed PD at 18 weeks [136]. The administration of the anti-GPC3-CAR-T cells has been associated with eradicating GPC3-positive HCC cells [154,155,156,157]. Moreover, other clinical trials have investigated monotherapies with anti-GPC3-CAR-T cells (NCT03980288, NCT04121273, and NCT03884751) [158,159,160], or in combination with cyclophosphamide and fludarabine (NCT02905188) [159].

### 5.6. Combination Therapy

The combination of different therapies for HCC patients showed promising results. The combination of two immunotherapies, or immunotherapies combined with conventional therapeutic approaches such as angiogenesis inhibitors, represents an important anti-HCC immune opportunity and has received significant attention.

An open-label, multicenter, multiarm, phase 1b study (NCT02715531) looked at the efficacy and safety of combining atezolizumab (anti-PD-L1 mAb) and bevacizumab (anti-VEGF mAb) in patients with unresectable HCC who had not previously received systemic therapy. The study found that atezolizumab plus bevacizumab improved PFS and reduced the risk of progression or death when compared to atezolizumab alone [161].

An open-label multicenter single-arm phase Ib study (NCT03006926) looked at the efficacy of combining lenvatinib (anti-VEGFR mAb) with pembrolizumab (anti-PD-1 mAb) in patients with unresectable HCC. Patients had a low toxicity profile and a high response rate due to an increase in antitumor activity. The FDA has labeled the treatment “revolutionary” for the first-line treatment of patients with unresectable HCC who are not candidates for other therapies [162].

A randomized, open-label, multicenter trial (NCT03794440) in China has shown the acceptable safety of the combination of sintilimab (anti-PD1 mAb) and bevacizumab (anti-VEGF mAb) as a first-line treatment in patients with advanced HCC. This study has revealed that sintilimab plus bevacizumab is associated with significantly better clinical benefits than sorafenib in patients with advanced HCC [163].

Furthermore, growing preclinical evidence has shown promising results in the combination of ICIs and CAR-T [164]. Guo et al. have used clustered regularly interspaced short palindromic repeats (CRISPR)/CRISPR-associated protein 9 (Cas9) technology to develop PD-1-knockout CAR-T cells. The CRISPR-mediated disruption of endogenous PD-1 can improve the CAR-dependent antitumor activity of the GPC3-specific second-generation CAR-T cells using CD28 as the costimulatory domain, as well as the in vivo persistence and infiltration of CAR-T cells, but has no effect on the CD4 and CD8 cells or the activation status of the CAR-T cells. In conclusion, the in vivo and in vitro results have shown that disrupting PD-1 can enhance the CAR-T cell-mediated antitumoral immune responses [165].

## 6. Single Cell RNA Sequencing in Cancer

In the last years, rapid advancements in next-generation sequencing (NGS) technologies have yielded valuable insights into cancer genetics. Indeed, scRNA-seq is a promising technology that can be applied to various research fields due to its ability to analyze rare cell populations, identify gene regulatory links, and follow the evolution of different cell lineages [166].

**Figure 3:**
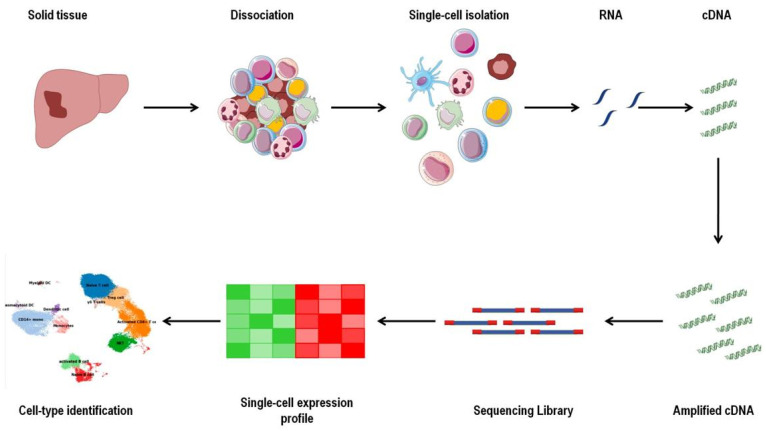
Workflow of single-cell RNA sequencing. The first step is the tissue dissociation into a cell suspension. The single-cell is lysed after isolation, and the RNA is collected and transcribed to cDNA via reverse transcription. After that, the cDNA is amplified to create a high-throughput RNA-seq library. The analysis of single-cell expression profiles permits the identification of different cell populations and subpopulations. The scRNA-seq allows for the characterizing of immune cells within the tumor microenvironment, as well as cells present in low numbers and in the quiescent state (https://smart.servier.com accessed on 23 August 2021).

Single cells must first be separated from each other for processing, which often involves tissue dissociation into a liquid cell suspension. Single cells are lysed after isolation, and the RNA within the individual cells is collected and transcribed to cDNA via reverse transcription. After that, the cDNA is amplified. Finally, high-throughput RNA-seq libraries are created in which the transcripts are mapped to the individual cells [18] (Figure 3). Indeed, the dissociation of the cells from the tissues is a fundamental step in applying scRNA-seq to tumor samples. Possible transcriptional alterations resulting from the sample collection and the processing could represent a limitation of this technology. Some researchers have attempted to address this technical issue by using cell lines or organoids [167,168].

scRNA-seq can demonstrate intertumoral and intratumoral heterogeneity that was not possible to study using bulk sequencing [18]. Moreover, multiple cell types belonging to distinct molecular patterns have been found in the tumor using scRNA-seq experiments. The presence of functionally diverse cell populations within the tumors might indicate their adaptation to the hostile tumor microenvironment. In this context, scRNA-seq technologies allow us to identify and analyze the subpopulations that are present in extremely low numbers and in a quiescent state, such as cancer stem cells (CSCs). CSCs are unique subpopulations of tumor cells that are resistant to conventional anticancer therapies and can reproduce tumor bulk after anticancer therapies [169]. Besides, the single-cell sequencing technologies can discover treatment-resistant cell subpopulations, thus allowing appropriate therapies to be chosen [170].

In addition, scRNA-seq experiments can allow for the characterizing of the immune cells within the tumor microenvironment [170,171,172]. Zheng et al. have investigated the entire TCR sequences and transcriptomes of >5000 single T cells isolated from HCC patients and identified 11 separate T cell subsets with different distribution patterns [63]. Indeed, single-cell sequencing technologies provide ample opportunities to investigate the origin of CTCs [170,173,174].

### Single-Cell Approach to Defining Immune Profile in HCC

The biology immune cells and the intercellular cross-talk in the HCC microenvironment are highly complicated [175,176]. Although HCC tumors include a high number of TILs, these TILs cannot destroy the tumor cells. The application of the scRNA-seq technologies can allow us to reveal distinct subtypes and the clonal expansion of TILs in HCC. Zhang et al. have used a combination of two single-cell RNA sequencing technologies, i.e., SMART-seq2 and droplet-based platforms, to study the landscape of the immune cells in HCC [177]. As a result, the combination of the analyses of these two technologies may provide us with a more comprehensive picture of the immune cells [178].

Zhang et al. have investigated immune cell composition, functional states, and cellular contacts in HCC tumors. The use of the two scRNA-seq technologies have allowed for the description of the immunological components of the HCC [177].

They have identified six macrophage clusters, and in particular, M4-c1-THBS1 and M4-c2-C1QA (TAM-like macrophages) have been abundant in tumor tissues. The genetic profile of M4-c1-THBS1 has been enriched for MDSCs signatures. These MDSC-like cells have demonstrated high levels of ficolin 1(FCN1) and versican (VCAN) but low levels of HLA-related genes. Besides, the expression of solute carrier family 40 member 1 (SLC40A1) and glycoprotein Nmb (GPNMB) have been elevated. SLC40A1 encodes ferroportin, an iron exporter, and modulates the TLR stimulus-induced proinflammatory cytokines, e.g., IL-6, IL-23, and IL-1β. This suggests that iron metabolism is involved in the innate immunity of the tumor microenvironment [177]. Another scRNA-seq-based study has shown that M2 macrophages are the predominant population of TAMs in HCC. Ho et al. have demonstrated that TAMs have high expression levels of two immunosuppressive molecules, i.e., leucocyte-associated immunoglobulin-like receptor 1 (LAIR1) and hepatitis A virus cellular receptor 2 (HAVCR2) (known as Tim-3) [17].

Zhang et al. have found that lysosomal-associated membrane protein 3 (LAMP3)^+^ DCs, which express the most number of ligands to bind the receptors of the T cells and the NK cells, can substantially regulate lymphocytes. They have also identified a remarkable association between the DCs and Treg, which might indicate the dysfunction of the DCs [177].

The scRNA-seq data have discovered several clusters of tumor-infiltrating CD8^+^ T cells. Although the exhausted CD8^+^ T cells have been the majority of the tumor-infiltrating CD8^+^ T cells, CX3CR1-and granzyme K (GZMK)-expressing CD8^+^ T cells have also been identified [179]. The analysis of the GZMK cluster has shown lower levels of cytotoxic markers, i.e., granzyme B (GZMB), granulysin (GNLY), and killer cell lectin-like receptor G1 (KLRG1), as well as specific exhaustion markers, i.e., PD-1, T cell immunoglobulin, and the ITIM domain (TIGIT). Blackburn et al. have shown that inhibiting the PD-1, the primary factor in T cell exhaustion, can decrease the apoptosis of the exhausted CD8^+^ T cells with intermediate PD-1 expression [180].

Mucosal-associated invariant T (MAIT) cells with semi-invariant TCR alpha chains are another cluster in HCC. The MAIT cells are activated in bacterial or viral infections and are thought to be the first line of defense in the liver; however, their role in cancer is unknown [179,181]. Zheng et al. have shown a remarkably decreased number of MAIT cells in tumors than in the neighboring normal liver tissues, which might support their antitumoral roles in HCC [63].

## 7. Conclusions

Immunotherapy is becoming one of the most important approaches for treating HCC. Combination therapies with ICIs, vaccines, oncolytic viruses, and conventional treatments at different stages of patients are one of the next prospects for immunotherapies to boost antitumor activity. Due to the heterogeneity of the tumor cells and the complexity of the immuno-regulatory mechanisms, multimodal immunotherapy treatments represent the next step in clinical antitumor efficacy, allowing researchers to advance the field and improve HCC patient outcomes. Furthermore, emerging technologies like single-cell RNA sequencing may help to determine the biomarkers of the predictive therapeutic response to, and maximize the efficacy of, immunotherapy.

## Figures and Tables

**Table 1:** Clinical trials and in vitro and in vivo studies on immunotherapy in HCC.

Regimen	Target	NCT	Design	Number of Patients	Clinical Results	Ref.
**ICIs**						
Nivolumab	PD-1	NCT01658878	Phase I/II	262	15–20% ORS, 15.6 months of overall survival OS, considerable tumor decrease	[129]
Pembrolizumab	PD-1	NCT02702414	Phase II	104	17% ORS, 1% CR, 16 PR, 44% stable disease SD, 33% progression	[130]
Camrelizumab	PD-1	NCT02989922	Phase II	220	14.7% ORS, 74.4% OS at 6 months, 55.9% OS at 12 months	[131]
Tislelizumab	PD-1	NCT03412773	Phase III	674	Recruiting	[131]
Durvalumab	PD-L1	NCT 01693562	Phase I/II	1022	Recruiting	[132]
Tremelimumab	CTLA-4	NCT01008358	Phase II	21	Partial response rate was 17.6%, and disease control rate was 76.4%. Time to progression was 6.48 months	[133]
Cabolimab	TIM-3	NCT02817633	Phase I	369	Recruiting	[110]
	**Agents**	**Descriptions**	**Design**	**Number of Patients**	**Clinical Results**	**Ref.**
**DC-Based Vaccine**						
	DCs pulsed with tumor cell lysate	Mature autologous DCs pulsed with HepG2 lysate	Phase I/II	33	2 patients had PR (13.3%), 9 patients had SD (60%), and 4 patients had PD (26.7%)	[134]
**Non-Cell-Based** **Vaccines**						
	A vaccine based on GPC3 peptides	Intradermal injections on days 1, 15, and 29	Phase I	33	1 patient had PR, 19 patients had SD, 9 patients decreased AFP, 30 patients’ specific CTL response	[135]
**ACT**						
	Anti-GPC3-CAR-T cells	GPC3-CAR treatment by intratumor injection	Phase I/II	4	2 patients SD, 1 patient PR after 4 weeks, and PD after 18 weeks	[136]
	**Agents**	**Descriptions**	**Design**	**Biological Effects**		**Ref.**
**DC-Based Vaccine**						
	DCs pulsed with NY-ESO-1	DCs pulsed with the recombinant NY-ESO-1	In vitro study	DCs loaded with NY-ESO-1 protein stimulate antigen-specific T cell responses against HCC cells in vitro.		[137]
**Non-Cell-Based** **Vaccines**						
	Injection of synthetic h-TERT	hTERT cDNA was subcloned in a plasmid	In vitro and in vivo study	6.9–12.5% of patients had an hTERT-specific CD8^+^T cell-mediated immune responses.		[138]
**OVs**						
	LDO-GFP	Herpes simplex virus type 1-based oncolytic vector-based oncolytic vector	In vitro and in vivo study	Decreased tumor growth		[139]
	GP73-SphK1sR-Ad5	Oncolytic adenovirus	In vitro and in vivo study	Apoptosis in HCC cells and decreased tumor volume		[140]
	VV-IL-37	Vaccinia virus expressing IL-37	In vitro study	Antitumoral immune responses		[141]

Abbreviation: ICI: immuno-checkpoint inhibitor; PD-1: programmed-death-1; PD-L1: programmed-death-ligand-1; CTLA-4: cytotoxic T-lymphocyte antigen 4; TIM3: mucin domain molecule 3; ORS: objective response rates; OS: overall survival; CR: complete response; PR: partial response; SD: stable disease; PD: progressive disease; IFN-γ: interferon-gamma; DC: dendritic cell; AFP: alpha-fetoprotein; NYESO-1: New York esophageal squamous cell carcinoma-1; GPC-3: glypican-3; hTERT: telomerase reverse transcriptase; GP73-SphK1sR-Ad5: Golgi protein 73-sphingosine kinase 1-short RNA-adenovirus serotype 5; VV: vaccinia virus; ACT: Adoptive cell transfer; CAR-T: chimeric antigen receptor T.

## Data Availability

Not available.
